# Expression of Human Thrombomodulin Prevents Early Thrombocytopenia and Thrombotic Microangiopathy in Pig‐to‐Nonhuman Primate Orthotopic Liver Xenotransplantation

**DOI:** 10.1111/xen.70120

**Published:** 2026-03-23

**Authors:** Zhongqiang Zhang, Ting Li, Qiang Li, Bin Xie, Xinger Zhao, Jianbin Wang, Yaxun Huang, Jing Luo, Shali Wen, Yinchun Zhou, Yong Deng, Yanfang Lu, Yanyan Jiang, Jiequn Li, David K. C. Cooper, Dengke Pan, Zhongzhou Si, Haizhi Qi

**Affiliations:** ^1^ Department of Liver Transplantation The Second Xiangya Hospital of Central South University Changsha Hunan China; ^2^ Department of Anesthesiology The Second Xiangya Hospital of Central South University Changsha Hunan China; ^3^ Operation Room The Second Xiangya Hospital of Central South University Changsha Hunan China; ^4^ Xenotransplantation and Regeneration Key Laboratory of Sichuan Province Chengdu ClonOrgan Biotechnology Co. Ltd Chengdu China; ^5^ Center For Transplantation Sciences Department of Surgery Massachusetts General Hospital/Harvard Medical School Boston Massachusetts USA

**Keywords:** liver, pig, rejection, thrombocytopenia, thrombotic microangiopathy, Tibetan macaque, xenotransplantation

## Abstract

**Background:**

Severe thrombocytopenia and coagulation dysregulation remain major barriers to survival in pig‐to‐primate liver xenotransplantation models.

**Methods:**

Porcine livers with five genetic modifications (GTKO, CMAHKO, β4GalNT2KO, hCD55, and hTBM) were orthotopically transplanted into four Tibetan macaques under two conventional immunosuppressive protocols. Longitudinal monitoring included graft and multi‐organ function, hematology, coagulation, and immune responses. Histopathological and immunohistochemical analyses were performed to characterize graft and recipient pathology.

**Results:**

High expression of human thrombomodulin (hTBM) in donor livers effectively prevented early rapid and severe thrombocytopenia, with three of four recipients maintaining platelets >100 × 10^9^/L. Thrombotic microangiopathy (TMA) was absent in grafts and recipient organs despite transient hypercoagulability. No spontaneous bleeding occurred in three of four cases. However, porcine livers failed to synthesize functional coagulation factor II, protein C, and protein S, contributing to progressive coagulopathy. While early graft function and coagulation remained stable for four posttransplant days, late dysfunction ensued, characterized by impaired coagulation factor synthesis and progressive graft failure. Antibody‐mediated rejection, accompanied by IgM/IgG/C4b deposition, anti‐TKO antibody increase, and pvWF upregulation, triggered injury that was amplified by innate and T cell–mediated responses.

**Conclusions:**

GTKO/CMAHKO/β4GalNT2KO/hCD55/hTBM donor liver transplantation was associated with an absence of early severe thrombocytopenia, transfusion requirement, and TMA, but long‐term survival was limited by immune‐mediated rejection and cross‐species incompatibilities in coagulation factor synthesis. Optimizing (i) donor genetic modifications and (ii) immunosuppressive therapy, with (iii) replacement of targeted coagulation factors will be crucial for achieving durable survival in pig liver xenotransplantation.

**Abbreviations**
AMR: antibody‐mediated rejection
CD55: decay accelerating factor
CMAHKO: Cytidine monophosphate‐N‐acetylneuraminic acid hydroxylase gene‐knockout
CVF: cobra venom factor
GTKO: α1,3‐galactosyltransferase gene‐knockout
LXT: liver xenotransplantation
NHP: nonhuman primate
PAEC: porcine aortic endothelial cell
PBMC: peripheral blood mononuclear cell
TBM: thrombomodulin
TCMR: T cell‐mediated rejection
TKO: triple knockout
TMA: thrombotic microangiopathy
WT: wild type
β4GalNT2KO: β‐1,4N‐acetylgalactosaminyltransferase 2 gene‐knockout

## 1 Introduction

Liver xenotransplantation (LXT) from genetically engineered pigs to humans offers a potential solution to the critical shortage of human donor organs [1, 2]. Advances in gene‐editing technologies and immunosuppressive regimens have enabled substantial progress in preclinical xenotransplantation models, particularly kidney and heart transplantation, where porcine grafts have supported survival for extended periods [3, 4]. More recently, these pig organs have been transplanted into humans [5, 6, 7, 8].

In contrast, pig‐to‐nonhuman primate (NHP) LXT remains challenging, with survival significantly shorter than in other organ models [9] with the exception of the lung. The major barriers include interspecies incompatibilities that manifest as severe thrombocytopenia, bleeding, and thrombotic microangiopathy (TMA), typically arising in the early postoperative period and profoundly limiting recipient survival [10, 11].

Genetic modifications of donor pigs targeting xenoantigens, complement pathways, and the coagulation cascade, combined with optimized immunosuppressive regimens, have emerged as the principal strategies to address these challenges [2, 12]. However, few studies have employed donor pigs incorporating all three categories of genetic modifications in preclinical orthotopic LXT. To date, only one reported case used a triple‐knockout (TKO) donor—deficient in GGTA1 (α‐1,3‐galactosyltransferase [Gal]), β4GalNT2 (β‐1,4‐N‐acetyl‐galactosaminyltransferase 2 [Sda]), and CMAH (N‐glycolylneuraminic acid [Neu5Gc] synthase)—combined with transgenic expression of human complement‐regulatory (hCD55) and coagulation‐related protein (hTBM). Paradoxically, this case achieved shorter survival than reports using donors with fewer genetic modifications, despite the use of comparable immunosuppressive regimens [13].

Thus, the optimal combination of donor genetic modifications—particularly high hTBM expression in the graft—required to prevent thrombocytopenia, coagulopathy, and immune‐mediated rejection in LXT remains to be determined.

In this study, we evaluated porcine livers with GTKO/CMAHKO/β4GalNT2KO/hCD55/hTBM modifications in a pig‐to‐NHP orthotopic LXT model under two distinct conventional immunosuppressive protocols. We systematically assessed graft and multi‐organ function, hematologic parameters, coagulation profiles, and immune responses to determine the efficacy of this combined strategy in mitigating recipient coagulopathy and thrombocytopenia, elucidating the mechanisms of graft failure, and identifying key factors limiting long‐term graft survival. Our findings provide critical insights into the current advantages and limitations of genetic and pharmacological strategies in LXT and highlight directions for future optimization.

## 2 Materials and Methods

### 2.1 Ethics

All animal care and experimental procedures complied with the Guide for the Care and Use of Laboratory Animals and were approved by the Independent Ethics Committee of the Second Xiangya Hospital, Central South University, and the Animal Research Institute of Sichuan Academy of Medical Sciences. The use of Tibetan macaques was specifically authorized by the Sichuan Provincial Forestry and Grassland Bureau.

### 2.2 Animals

#### 2.2.1 Donor Pigs

Genetically engineered male Bama miniature pigs (6.0–10.0 kg, blood type O) were used as donors (Table 1). Pigs carried combined genetic modifications: GGTA1 knockout (GTKO), CMAH knockout (CMAHKO), β4GalNT2 knockout (β4GalNT2KO), and transgenes encoding human CD55 (hCD55) and thrombomodulin (hTBM) (ClonOrgan Biotechnology Co., Ltd., Chengdu, China). Genotypes were confirmed by PCR and sequencing. Expression of target proteins and absence of xenoantigens were verified by flow cytometry on porcine aortic endothelial cells (PAECs) pretransplant and by immunofluorescence on graft tissue posttransplant.

**TABLE 1:** Details of donor and recipient in orthotopic LXT.

Recipient ID	Donor ID	Donor age (days)	Graft weight (g)	Donor weight (Kg)	Recipient age (years)	Recipient liver weight (g)	Recipient weight (Kg)	Survival (days)	Clinical outcome
M335425	P3993	63	240	7.6	16	261	16.9	5	Pneumonia, Sepsis, MODS
M823808	P4442	57	177	6.8	16	260	15.8	5	MODS
M335304	P4698	64	240	7.8	18	232	14.45	3	MODS; IVC and partial PV thrombosis caused by surgical procedure
M335244	P5294	67	250	9.9	13	292	18.3	7	MODS

^a†^ Donor pigs bearing five combined genetic modifications: GTKO, CMAHKO, β4GalNT2KO, human CD55, and thrombomodulin (TBM); MODS = Multi‐Organ Dysfunction Syndrome; IVC = inferior vena cava; PV = portal vein.

#### 2.2.2 Recipient Nonhuman Primates

Four healthy, captive‐bred male Tibetan macaques (14.0–19.0 kg) were used as recipients (Table 1; Sichuan Academy of Medical Sciences, Chengdu, China). Recipients were screened for low serum levels of anti‐donor pig IgM/IgG and minimal complement‐dependent cytotoxicity (CDC). All animals were confirmed negative for simian immunodeficiency virus, simian T‐cell lymphotropic virus, simian retrovirus, and hepatitis B virus.

### 2.3 Immunosuppressive Regimens and Adjunctive Therapy

Two Perioperative Immunosuppressive Regimens Were Employed (Table 2)

**TABLE 2:** Immunosuppressive regimens.

Recipient		Drug	Dose	Schedule
**M335304** **M823808** **M335425** ***(Protocol‐1)***	*Induction*	Anti‐thymocyte globulin (ATG)	2.5 mg/kg i.v.	Days ‐3 and ‐2
		Cobra venom factor (CVF)	100 units/kg i.v.	Days 0 and 1
			
	*Maintenance*	Tacrolimus (FK‐506)	0.02–0.04 mg/kg i.m. (maintain level of 10–15 ng/ml)	Daily from day ‐2
		Methylprednisolone	20 mg/kg i.v. on day 0–3, then tapered to 1.25 mg/kg	Daily from day 0
		Tocilizumab	10 mg/kg i.v.	Day ‐3
		Etanercept	0.5 mg/kg s.c.	Days 0, 1, 3
**M335244** ***(Protocol‐2)***	*Induction*	Rituximab (anti‐CD20 mAb)	10 mg/kg i.v.	Day ‐5
		Anti‐thymocyte globulin (ATG)	2.5 mg/kg i.v.	Days ‐3 and ‐2
		Cobra venom factor (CVF)	100 units/kg i.v.	Days 0 and 1
			
	*Maintenance*	Tacrolimus (FK‐506)	0.02–0.04 mg/kg i.m. (maintain level of 10–15 ng/ml)	Daily from day ‐2
		Methylprednisolone	20 mg/kg i.v. on day 0–3, then tapered to 1.25 mg/kg	Daily from day 0
		Etanercept	0.5 mg/kg s.c.	Days 0, 1, 3

^†^Day 0 = surgery day

#### 2.3.1 Protocol 1 (M335302, M335304, M335298)

Induction therapy consisted of anti‐thymocyte globulin (Genzyme, Cambridge, MA, USA) and cobra venom factor (Quidel Corp., A600, San Diego, CA, USA). Maintenance immunosuppression included tacrolimus (Astellas Pharma, Tokyo, Japan) and corticosteroids (methylprednisolone, Solu‐Medrol, Pfizer, New York, USA). In addition, tocilizumab (anti–IL‐6 receptor monoclonal antibody, Roche Pharma, Basel, Switzerland) and etanercept (TNF‐α inhibitor, Enbrel, Pfizer, New York, USA) were administered.

#### 2.3.2 Protocol‐2 (M335244)

This regimen was identical to Protocol‐1, except that tocilizumab was omitted, and a single dose of anti‐CD20 monoclonal antibody (rituximab, Roche Pharma, Basel, Switzerland) was added preoperatively.

All recipients received prophylactic antibiotics and ganciclovir for infection prevention. Human prothrombin complex concentrate (hPCC; Hualan Biological Engineering Co., Ltd., China), containing coagulation factors II, V, VII, and IX, was administered as a single intravenous dose (20 IU/kg) whenever coagulation abnormalities were detected.

### 2.4 Orthotopic LXT

Donor pigs were anesthetized with Zoletil (10 mg/kg i.m.) and maintained with isoflurane (1–2%). Following systemic heparinization (200 U/kg), the liver was harvested with standard technique under cold perfusion with lactated Ringer's solution (500 mL, via the abdominal aorta), followed by University of Wisconsin (UW) solution (500 mL, via the portal vein). Because UW solution is relatively viscous, the liver was not well perfused when we only used UW solution for perfusion. Cold ischemia time was <3 h in all experiments.

Recipient monkeys were pre‐medicated with atropine (0.01 mg/kg i.m.) and anesthetized with Zoletil (10 mg/kg i.m.), intubated, and maintained with isoflurane. Orthotopic transplantation was performed using standard end‐to‐end vascular anastomoses (suprahepatic and infrahepatic vena cava, portal vein, and hepatic artery). In recipient M335244, external biliary drainage with a T‐tube was established; all others underwent end‐to‐end bile duct anastomosis. Portal vein reperfusion preceded infrahepatic vena cava and hepatic artery reperfusion.

### 2.5 Flow Cytometry and Immunologic Assays

To confirm donor genotypes, porcine aortic endothelial cell (PAECs) from donor pigs were analyzed by flow cytometry for expression of Gal, Sda, Neu5Gc, hCD55, and hTBM. Circulating anti‐TKO pig antibodies (IgM/IgG) in recipient serum were measured postoperatively as previously described [14].

### 2.6 Sample Collection and Clinical Monitoring

Peripheral blood was collected preoperatively, 2 h post‐reperfusion, and daily until endpoint. Complete blood count, serum biochemistry, coagulation parameters, and complement C3/C4 levels were analyzed (Wuhou Lab & Health Inspection, Chengdu, China). Arterial lactic acid was measured using a blood gas analyzer (Seamaty, Chengdu, China). Coagulation dynamics in M335244 were assessed using a Sonoclot analyzer (Viscell Medical Group, USA). Plasma coagulation factors were measured with an automated coagulation analyzer (STAR MAX, STAGO, France). Bile output was quantified daily in M335244.

### 2.7 Histology and Immunohistochemistry

Liver tissue samples were collected pretransplant, 2 h post‐reperfusion, and at necropsy; other major organs were sampled at necropsy. Samples were fixed in 10% neutral‐buffered formalin, paraffin‐embedded, and sectioned (4 µm). Hematoxylin and eosin (H&E) staining was used for histopathology. Immunohistochemistry was performed for complement deposition (IgM [Dako‐Agilent], IgG [AB2337528], C4b [AB136921]), immune cell infiltration (CD68 [GM081404], CD11b [TA807952], CD3 [ZF0301]), and endothelial activation (pvWF [AB6994]). Immunofluorescence was performed to verify donor genotype (BSI‐B4 [L2895‐1], DBA [FL‐1031‐5], Neu5Gc [Poly21469], hCD55 [BRIC 216], hTBM [SC‐13164]). Sections were examined by two blinded pathologists, and positive staining was quantified using ImageJ software.

### 2.8 Statistical Analysis

Data are presented as Mean ± Standard Deviation (SD) or Median where appropriate. Comparisons between groups were performed using Paired *t*‐test or One‐way ANOVA Test. A *p*‐value < 0.05 was considered statistically significant. Analyses were performed with GraphPad Prism (version 10.5.0)

## 3 Results

### 3.1 Donor genetic Modifications, Perioperative Management Strategies, and Recipient Outcomes

**FIGURE 1:**
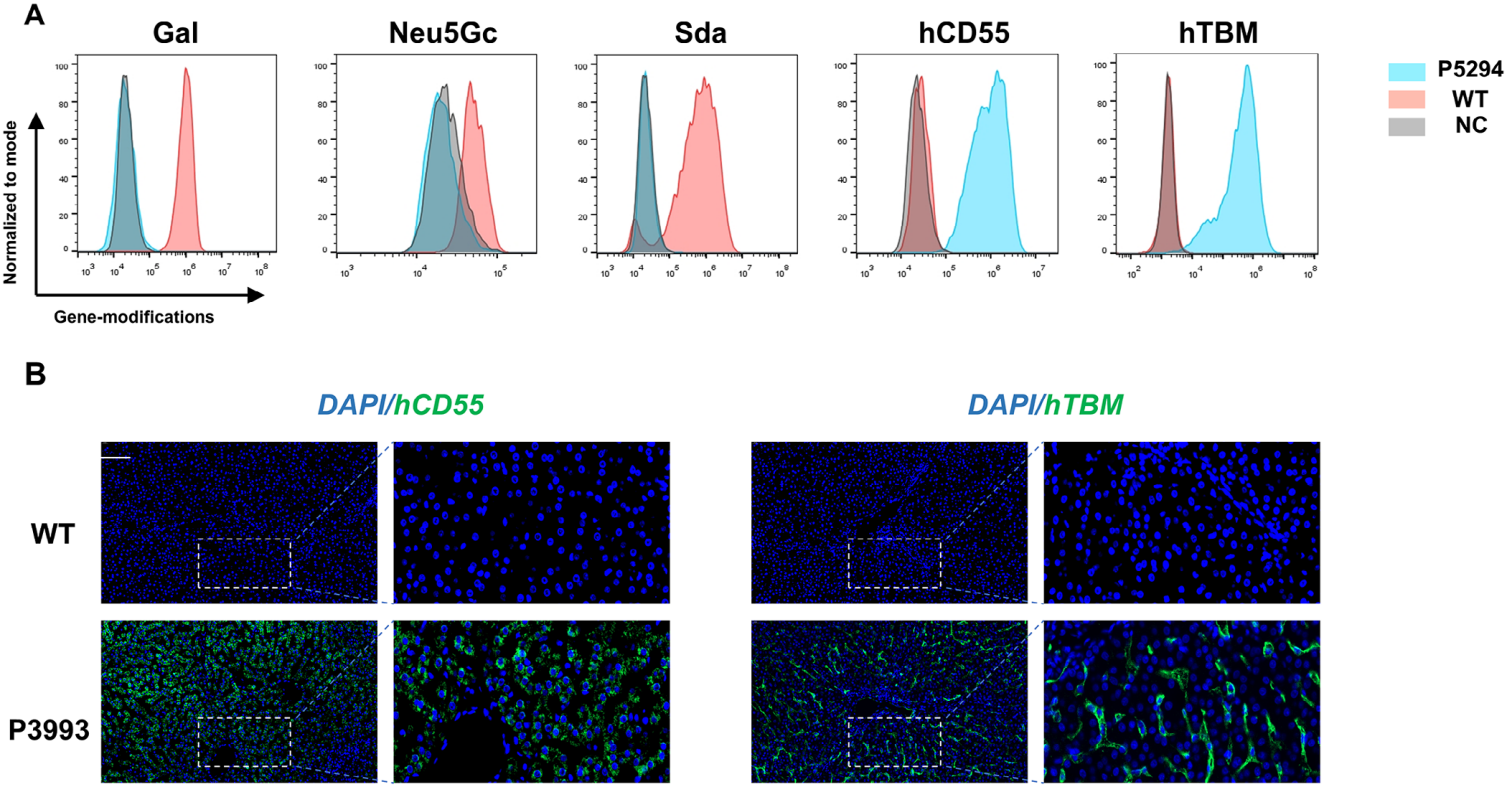
Donor genetic modifications (A) Flow cytometry analysis of porcine aortic endothelial cells (PAECs) from donor P5294 and wild‐type (WT) pig showing expression of Gal, Neu5Gc, Sda, hCD55, and hTBM. NC = negative control. (B) Immunofluorescence staining of donor liver tissue demonstrating expression of human CD55 and thrombomodulin (TBM) in P3993 compared with WT pig. Positive signals are shown in green (400× magnification, boxed regions further magnified 10×). WT = wild type pig.

Prior to transplantation, donor PAECs genotypes were confirmed by flow cytometry, with representative results from donor P5294 shown in Figure 1A. Postoperatively, genotypes were reconfirmed in donor liver tissue by immunofluorescence (Figure 1B). The three major xenoantigen genes—GGTA1 (Gal), β4GalNT2 (Sda), and CMAH (Neu5Gc)—were completely knocked out, preventing hyperacute rejection.

Furthermore, hCD55 and hTBM were robustly expressed on donor PAECs and graft tissue, conferring protection against complement activation and dysregulated coagulation.

Perioperative immunosuppressive protocols are summarized in Table 2. Recipient M335244 received a modified regimen, with reduced tocilizumab and a single dose of rituximab administered on postoperative day‐5.

The median survival time of recipients was 5 days. The primary cause of mortality was multi‐organ dysfunction syndrome (MODS), including graft failure, rather than massive hemorrhage, which has been the predominant cause of death in previous studies (Table 1).

### 3.2 Early preservation of Liver Function Followed by Progressive Impairment

**FIGURE 2:**
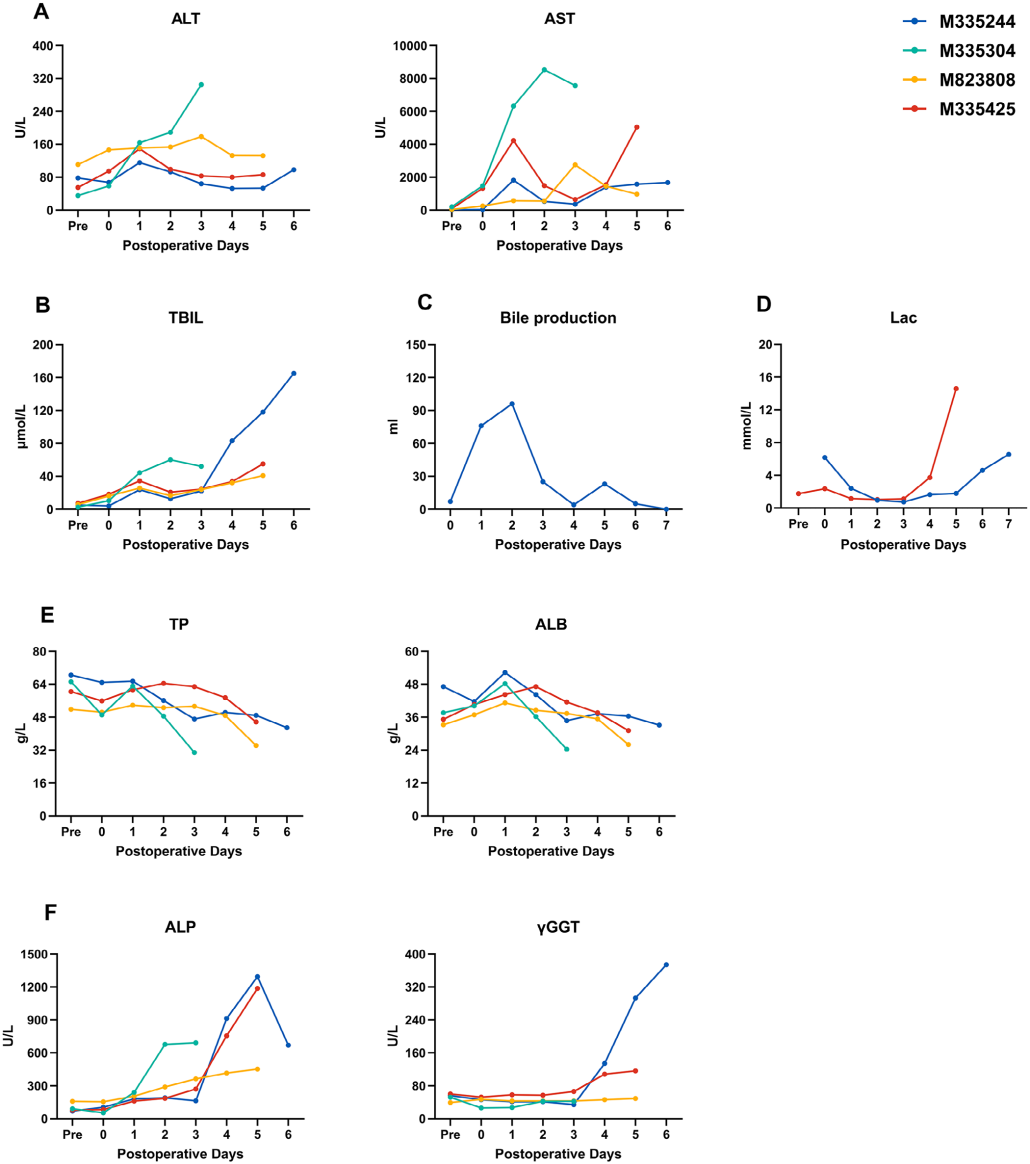
Functional parameters of liver xenografts (A) Alanine aminotransferase (ALT) and aspartate aminotransferase (AST).(B) Total bilirubin (TBIL).(C) Bile output in recipient M335244.(D) Blood lactate levels in M335244 and M335425. (E) Serum total protein (TP) and albumin (ALB). (F) Alkaline phosphatase (ALP) and γ‐glutamyl transferase (γGGT). Pre = preoperative baseline.

To assess graft function, clinical markers of hepatic injury and synthetic activity were serially monitored. In all recipients except M335304, alanine aminotransferase (ALT) and aspartate aminotransferase (AST) exhibited a biphasic pattern: an early postoperative rise, partial recovery, and subsequent elevation in later stages (Figure 2A). Recipient M335304 showed persistently high transaminase levels, attributable to thrombosis in branches of the infrahepatic inferior vena cava (IVC) and portal vein (PV) caused by surgical manipulation.

Serum total bilirubin (TBIL) paralleled ALT/AST kinetics (Figure 2B). In M335244, external biliary drainage permitted quantitative assessment of bile production. Bile secretion began shortly after portal vein reperfusion, peaked within 48 h, and subsequently declined to <30 mL/day (Figure 2C).

Blood lactate levels in M335244 and M335425 remained stable and low during the first 4 postoperative days, indicating preserved metabolic activity (Figure 2D). Serum total protein (TP) and albumin (ALB) were initially stable, supported by exogenous supplementation, but progressively declined with graft dysfunction, leading to ascites and subcutaneous edema (Figure 2E).

Markers of biliary integrity, alkaline phosphatase (ALP) and gamma‐glutamyl transferase (γGGT), remained stable initially but increased in the later stages, consistent with graft injury (Figure 2F).

### 3.3 Prevention of Severe Thrombocytopenia and Coagulation Dysregulation Without Transfusion

**FIGURE 3:**
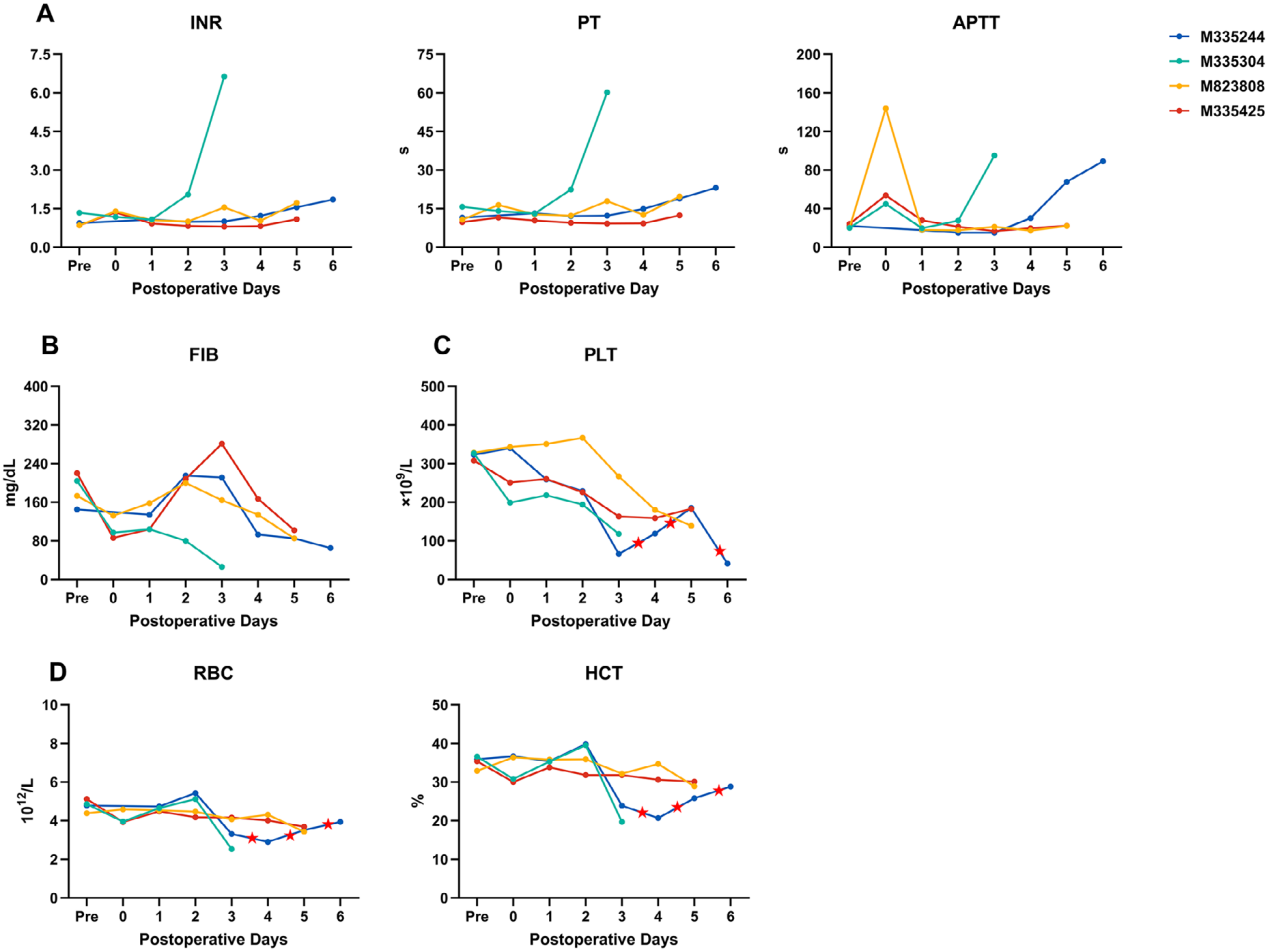
Coagulation profile and hematology International normalized ratio (INR), prothrombin time (PT), and activated partial thromboplastin time (APTT). (B) Plasma fibrinogen (FIB). (C) Platelet counts (PLT). (D) Red blood cell (RBC) counts and hematocrit (HCT). Pre = preoperative baseline. Red stars indicate blood transfusions for M335244.

Recipients (excluding M335304) maintained preserved coagulation profiles, with international normalized ratio (INR), prothrombin time (PT), and activated partial thromboplastin time (APTT) within acceptable ranges (Figure 3A). Serum fibrinogen levels were consistently elevated, suggesting active synthesis by porcine grafts (Figure 3B).

Importantly, unlike prior studies, rapid and severe thrombocytopenia did not occur. Platelet counts remained >100 × 10^9^/L in all recipients except M335244, without transfusion requirements (Figure 3C).

No spontaneous postoperative bleeding was observed, and red blood cell (RBC) counts and hematocrit (HCT) remained stable (Figure 3D). The exception was M335244, who developed massive subcutaneous hemorrhage from improper catheter management on postoperative day 3, requiring emergent transfusion. Subsequent persistent anemia of unclear etiology necessitated repeated transfusions (red stars in Figures 3C, D).

### 3.4 Human Prothrombin Complex Concentrates (hPCC) Improved Coagulation and Platelet Function

**FIGURE 4:**
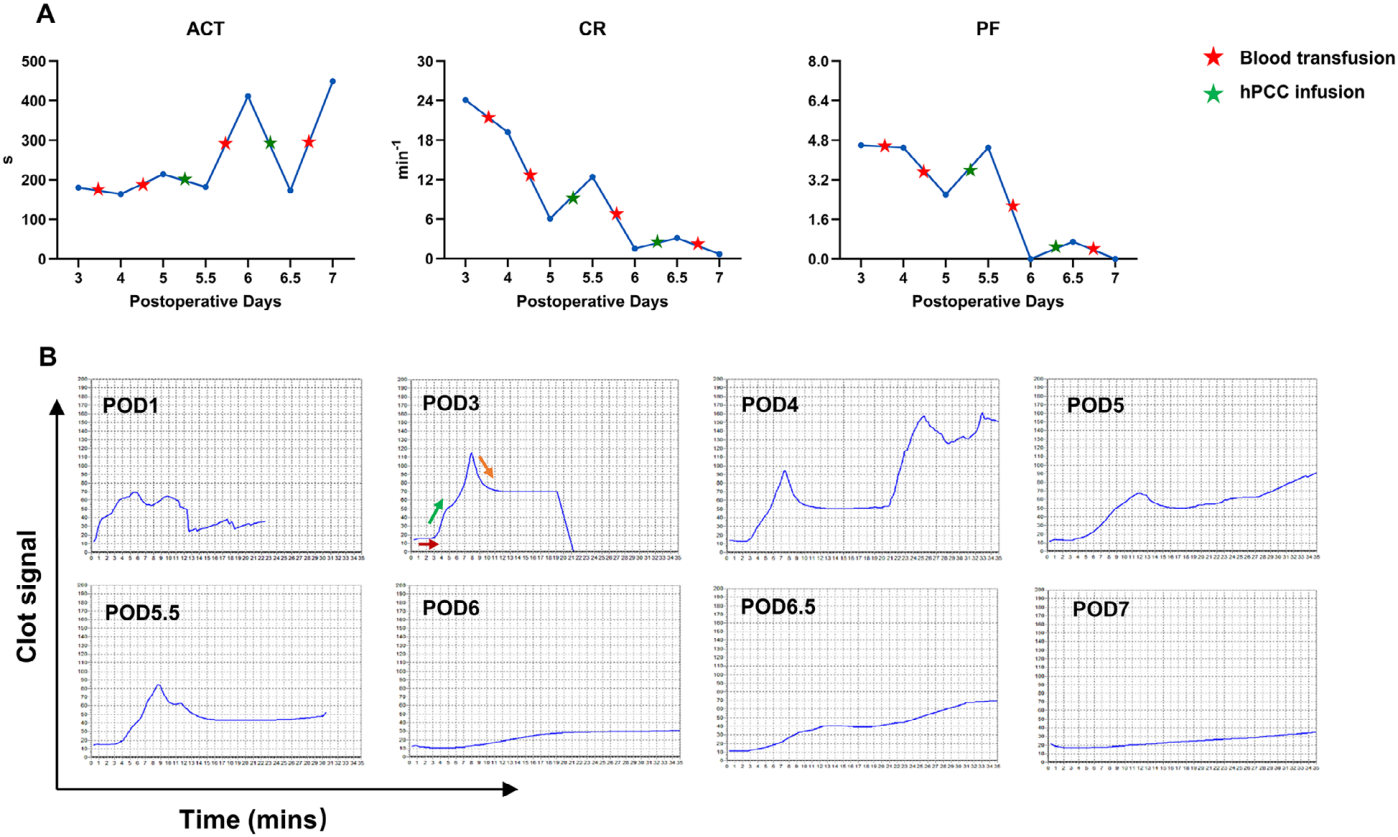
Coagulation dynamics in M335244 assessed by Sonoclot analyzer (A) Quantitative assessment of activated clotting time (ACT, normal range, 100–240 s, time to initial fibrin formation), platelet function (PF, normal range 1.0‐4.5, contribution of platelets to clot firmness, independent of platelet count), and clotting rate (CR, normal range 10.0–35.0 min^−1^, percentage of the peak amplitude per unit time, reflecting fibrinogen‐to‐fibrin conversion). ACT reflects global clotting factor activity. CR indirectly reflects fibrinogen levels. PF reflects platelet function. Red stars on line indicate blood transfusions; green stars on line indicate administration of human prothrombin complex concentrate (hPCC). (B) Sonoclot signatures from serial postoperative blood samples. In the signature graphs, ACT is indicated by the initial short line, CR by the gradient of the primary slope and PF by the third downward slope after the peak (all shown representatively in the POD3 graph, with red, green, and yellow arrows, respectively). Graphs marked POD3, POD4 and POD5.5 displayed normal or near‐normal Sonoclot signatures. In contrast, Graph marked POD1 showed a nearly absent initial short line and a rounded peak on Sonoclot signature, indicating a hypercoagulable state, whereas the remaining graphs showed markedly extended initial lines and flattened Sonoclot signatures, consistent with hypocoagulability (See references 37 and 38). POD = postoperative day.

Sonoclot analyzer was used to evaluate coagulation dynamics, including activated clotting time (ACT), clotting rate (CR), and platelet function (PF) (Figure 4). In M335244, early postoperative assessments suggested a hypercoagulable state, precluding accurate readings on day 1 (Figure 4B, POD1). By day 3, parameters normalized (Figure 4B, POD3–4). After blood transfusion (red star), ACT prolonged while CR and PF declined, indicating worsening coagulopathy (Figure 4A). Although transfusion temporarily increased HCT and platelet counts (Figures 3C, D), it paradoxically exacerbated platelet dysfunction (red stars, Figure 4A). In contrast, targeted hPCC infusion effectively restored both coagulation and platelet function (green stars, Figure 4A).

### 3.5 Porcine Liver Failed to Sustain Synthesis of Functional Coagulation Factor II, Protein C, and Protein S

**FIGURE 5:**
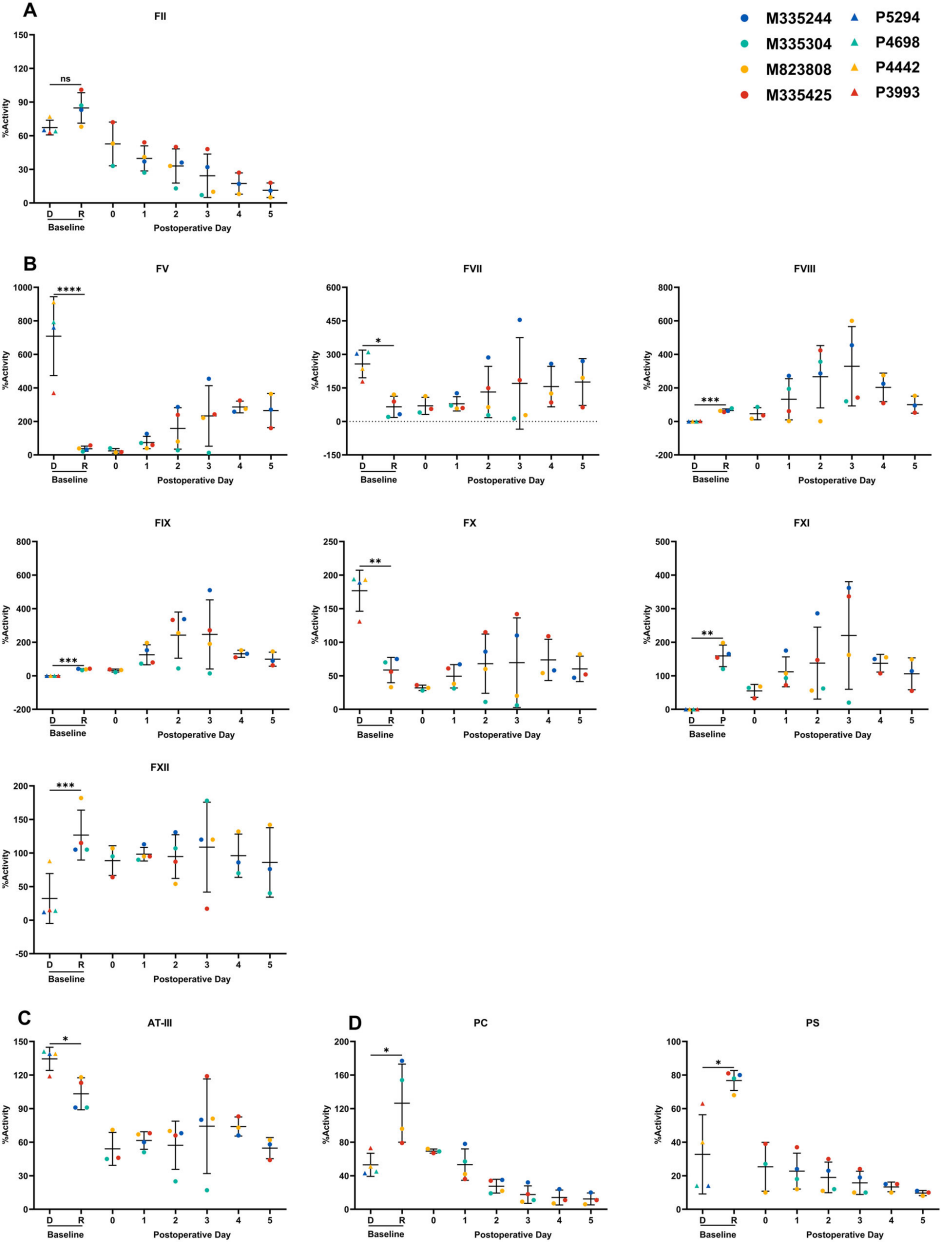
Circulating functional coagulation factors in donors and recipients (A) Coagulation factor II. (B) Factors V, VII, VIII, IX, X, XI, XII. (C) Antithrombin‐III (AT‐III). (D) Protein C (PC) and protein S (PS). D = donor; R = recipient. Baseline values were obtained from plasma collected pretransplant. *p < 0.05; **p < 0.01; ***p < 0.005.

Baseline assays revealed donor pigs had comparable FII activity to NHPs, higher FV, FVII, FX, and antithrombin‐III (AT‐III), but markedly lower FXII, protein C (PC), and protein S (PS). Donor pigs exhibited negligible FVIII, FIX, and FXI activity (Figure 5). Posttransplant, recipients initially showed increased activities of most coagulation factors, indicating functional porcine synthesis. However, FII, PC, and PS steadily declined, reflecting sustained synthesis defects (Figures 5A, D). Later, activities of other factors also decreased, correlating with graft dysfunction. These species‐specific deficiencies likely contributed to progressive coagulopathy

### 3.6 Absence of TMA and Hemorrhage, With Preserved Architecture

**FIGURE 6:**
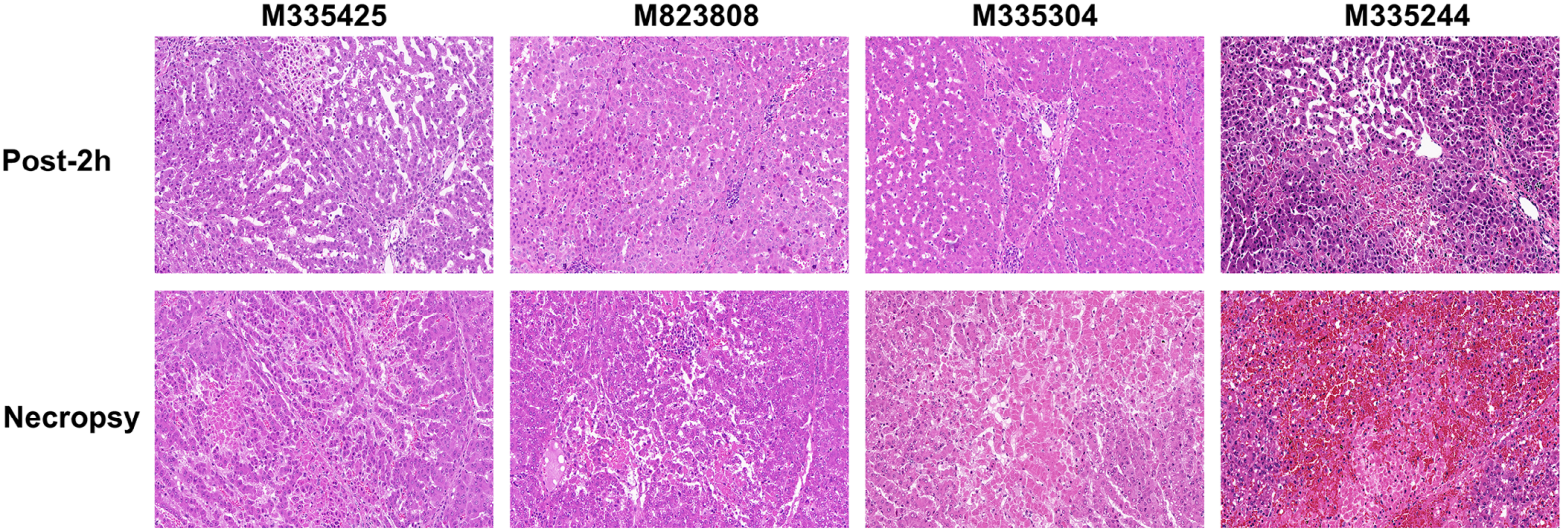
Histology of liver grafts Representative hematoxylin and eosin (H&E, ×400) staining of graft tissue at Post‐2 h and necropsy. Post‐2 h = 2 h post‐reperfusion; Necropsy = endpoint at necropsy.

At 2 h post‐reperfusion, H&E staining revealed intact lobular structure with minimal necrosis. At necropsy, focal centrilobular necrosis was evident, but overall architecture was preserved. Crucially, no TMA or hemorrhage was detected in grafts (Figure 6) or recipient organs (Figure S1). Only M335244 displayed marked sinusoidal erythrocyte congestion, likely due to repeated transfusions (Figure 6).

### 3.7 Immune‐mediated Injury Caused Progressive Dysfunction and Shortened Survival

#### 3.7.1 Antibody‐Mediated Rejection (AMR)

**FIGURE 7:**
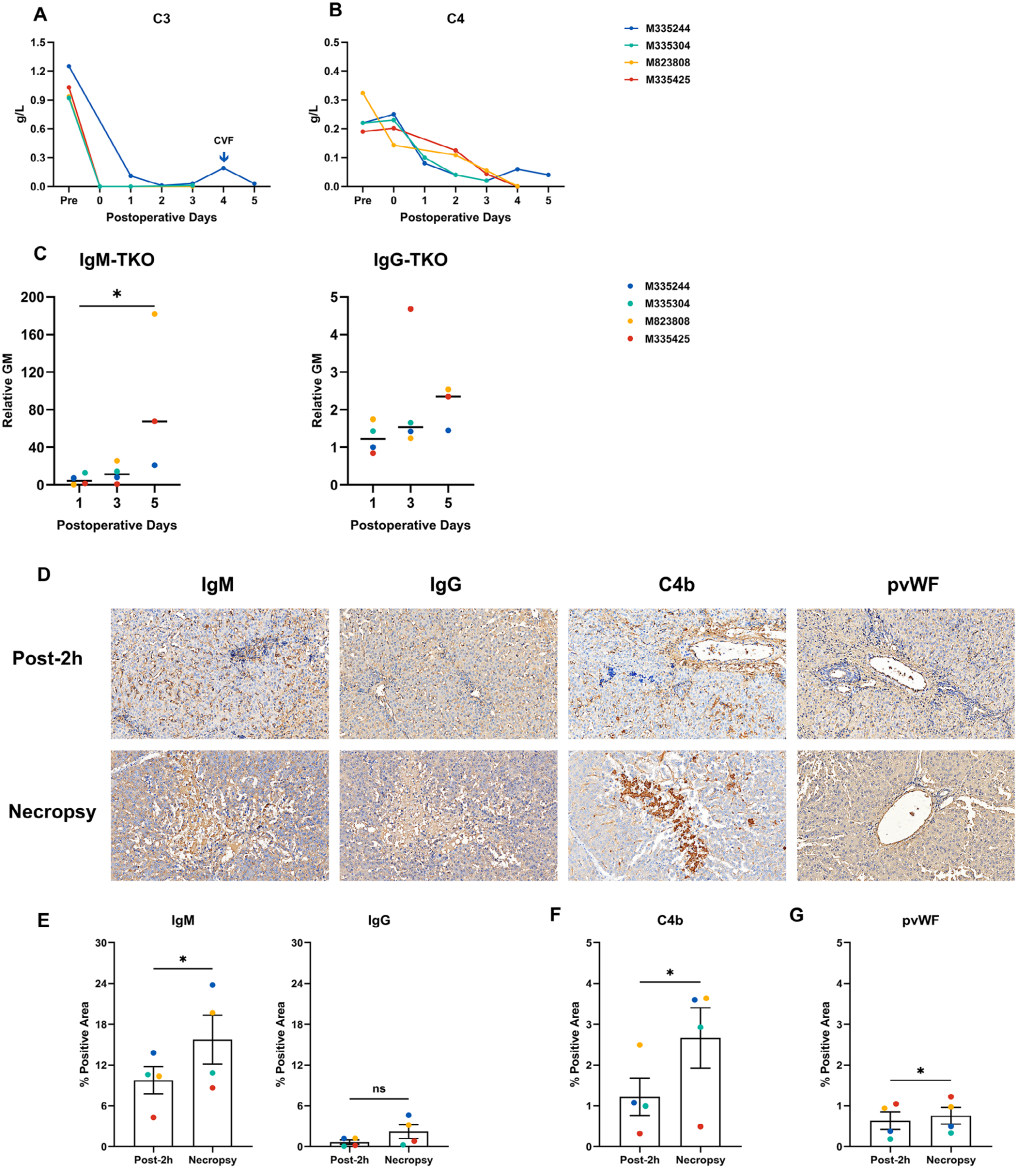
Antibody‐mediated rejection (A) Serum complement 3 (C3) and (B) complement 4 (C4) levels in recipients. (C) Serum IgM and IgG binding to TKO pig PBMCs over time. (D) Immunohistochemistry of graft tissue from recipient M823808 showing deposition of IgM, IgG, C4b, and pig von Willebrand factor (pvWF) at Post‐2 h and necropsy. (E) Quantification of IgM and IgG staining, (F) C4b staining, and (G) pvWF staining in high‐power fields (HPFs, ×400) at Post‐2 h and necropsy. Blue arrow indicates CVF administration; Post‐2 h = 2 h post‐reperfusion; Necropsy = endpoint at necropsy. *p < 0.05; ns = not significant.

Triple knockout (GTKO, CMAHKO, β4GalNT2KO) pigs with hCD55 and hTBM as donors were designed to mitigate AMR, and recipients received cobra venom factor (CVF). Except for M335244, preoperative C3 was effectively depleted (Figure 7A). Nonetheless, substantial IgM/IgG deposition occurred within graft vasculature as early as 2 h post‐reperfusion and persisted at necropsy (Figures 7D, E). Declining C4, strong C4b deposition, and rising anti‐TKO IgM/IgG confirmed complement activation (Figures 7B–E). Endothelial injury was further indicated by increased porcine vWF expression (Figures 7D, G). Pathology was most severe in M335244, that had incomplete C3 depletion and repeated transfusions, with C3 rebound on POD4. A second CVF dose transiently reduced C3 and restored bile output (Figures 2C, 7A), supporting complement‐driven rejection.

#### 3.7.2 Cellular Rejection

**FIGURE 8:**
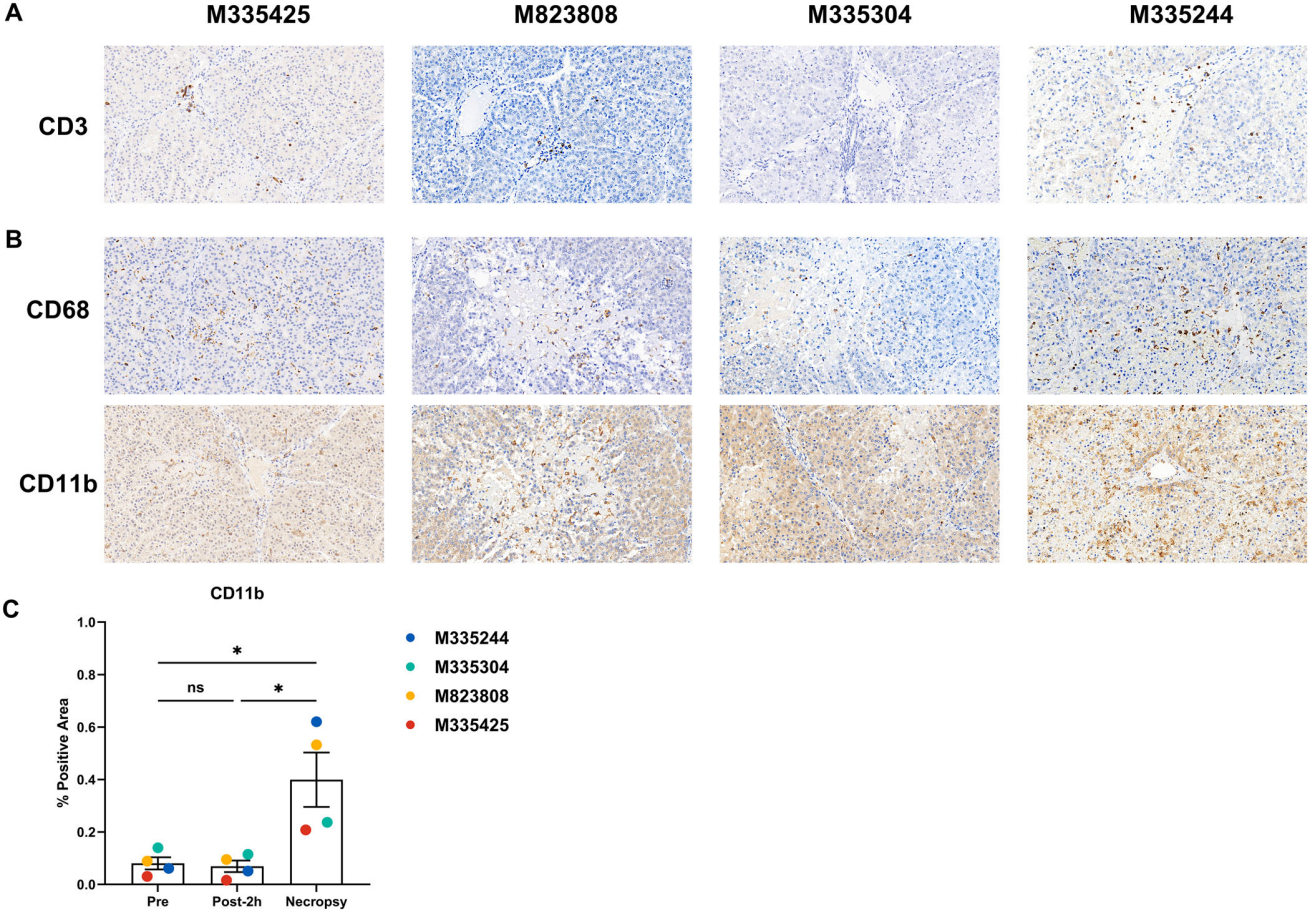
Cellular rejection Immunohistochemistry of graft tissue showing CD3 staining, (B) CD68 and CD11b staining at necropsy (×400). (C) Quantification of CD11b‐positive staining area in HPFs (×400) at multiple time points. Pre = preoperative baseline; Post‐2 h = 2 h post‐reperfusion; Necropsy = endpoint at necropsy. *p < 0.05; ns = not significant.

ATG induction suppressed early T‐cell activity, but lymphocyte counts rebounded later (Figure S2). CD3+ T cells were absent before and immediately after reperfusion, but infiltrated grafts at necropsy in most recipients, consistent with late‐onset TCMR (Figure 8A). Recipient‐derived CD68+ macrophages and CD11b+ myeloid cells infiltrated necrotic regions, indicating a major role of innate immune activation in graft injury (Figures 8B, C).

## 4 Discussion

Building on groundbreaking long‐term survival records from life‐supporting pig kidney and heart xenotransplantation trials in nonhuman primates [3, 4], genetically modified pig heart and kidney transplants in humans have been performed with moderate success [5, 8]. However, compared to kidney and heart transplantation, LXT remains particularly challenging, with significantly shorter survival times in pig‐to‐NHP models despite advanced genetic modifications.

The use of genetically engineered pigs in LXT was first reported in 2000, when Ramirez et al. employed pigs expressing human complement regulatory proteins (CD55 or CD55/CD59/H‐transferase) as donors [15, 16]. Compared with wild‐type pigs, these modifications markedly prolonged recipient survival by suppressing hyperacute rejection. Subsequent studies using GTKO pigs or GTKO/hCD46 pigs combined with optimized immunosuppressive regimens further reduced hyperacute rejection and significantly prolonged the survival in LXT [17]. Nevertheless, lethal coagulopathy—characterized by rapid thrombocytopenia, bleeding, and TMA—remained the major barrier limiting survival in pig‐to‐NHP LXT [10, 18, 19].

Severe thrombocytopenia has been attributed to excessive platelet activation/aggregation and aberrant sequestration/phagocytosis within porcine liver sinusoidal endothelial cells (LSECs), Kupffer cells, and hepatocytes [10, 20, 21]. Endothelial expression of porcine von Willebrand factor (pvWF) exacerbates platelet activation and aggregation [22, 23], whereas targeted disruption of platelet‐activation pathways has been shown to reduce platelet consumption [24, 25].

Another critical barrier lies in species incompatibilities of the thrombin–thrombomodulin (TM) complex. Porcine thrombomodulin can bind to human thrombin but fails to effectively activate protein C (PC), a key anticoagulant that inhibits factors Va and VIIIa [26]. In vitro, expression of human thrombomodulin (hTBM) on pig endothelial cells markedly reduced platelet aggregation and supported protein C activation [27], suggesting a potential solution to coagulation dysregulation. Indeed, in kidney and heart xenotransplantation models, hTBM expression has been associated with the absence of consumptive coagulopathy or thrombocytopenia [4, 28, 29].

In our study, we demonstrated for the first time in vivo that high hTBM expression prevented the rapid and severe thrombocytopenia typically observed in pig‐to‐NHP orthotopic LXT. With the exception of recipient M335244, all recipients maintained platelet counts above 100 × 10^9^/L without transfusion throughout survival. This protective effect of hTBM was consistent with recent results from a genetically‐modified pig‐to‐human decedent liver transplant, in which hTBM expression supported circulatory homeostasis [30]. However, in a 2023 LXT study [13], the benefit of hTBM was less apparent—highlighting that donor and recipient background, graft expression levels, and perioperative conditions may critically influence outcomes.

Despite these improvements, early postoperative grafts still exhibited endothelial activation, as evidenced by pvWF expression, likely triggered by ischemia–reperfusion injury and/or natural antibody binding. This contributed to a transient hypercoagulable state during the first two postoperative days, consistent with our Sonoclot analysis, although no TMA was detected in grafts or recipient organs. The absence of TMA may reflect the inhibitory effect of hTBM on the coagulation cascade of the recipient. According to the results of circulating coagulation parameters and the absence of TMA in the graft and the recipient's organs, the hTBM primarily protects the graft from the effect of the recipients’ coagulation cascade and then indirectly affects recipient's systemic coagulation.

Due to absence of TMA or spontaneous bleeding, no blood transfusion was required in any recipient except in M335244 (following improper arterial catheter management that resulted in a massive subcutaneous hemorrhage), characterized by stable levels of HCT and RBC count. We presume that the requirement for RBCs after LXT reported by previous studies can possibly be attributed to coagulation dysregulation leading to the consumption of RBCs.

Supplementation with human prothrombin complex concentrates (hPCC) effectively corrected coagulation dysregulation, and possibly helped platelet recovery through reduced consumption of platelets, thus achieving hemostasis [31, 32]. Nevertheless, indiscriminate administration—especially early postoperatively during a hypercoagulable phase—risked exacerbating coagulopathy and should be approached cautiously. Our data also suggest that targeted transfusion with hPCC improved coagulation and platelet function more successfully than whole‐blood transfusion, which transiently improved hematologic parameters but paradoxically worsened coagulopathy. This paradoxical deterioration may reflect whole‐blood transfusion‐associated immune amplification and persistent interspecies incompatibility within the coagulation regulatory axis, thereby aggravating endothelial activation and consumptive coagulopathy.

However, the administration of hPCC still failed to prevent the rapid and severe thrombocytopenia that occurred early after operation and required transfusions as well as the risk of TMA in the graft [31, 32].

Interestingly, our donor pigs exhibited no detectable baseline activity of FVIII, FIX, and FXI; however, these factors were synthesized by the grafts after transplantation, suggesting potential cross‐species functionality that warrants further investigation. In contrast, the grafts consistently failed to synthesize functional coagulation factor II, protein C, and protein S in recipients, highlighting fundamental species incompatibilities in coagulation factor synthesis.

Furthermore, the activity of coagulation factors synthesized by porcine grafts in recipients showed an initial postoperative increase, often exceeding the recipient's baseline levels, but subsequently declined in parallel with progressive graft dysfunction.

Notably, plasma ATT‐III activity never recovered to baseline; we therefore presume that these anticoagulants including PC and PS, together with hTBM, were being consumed in the process of counteracting thrombosis within the graft.

Immunologic injury emerged as another major barrier. Despite TKO and hCD55 modifications with preoperative CVF administration, grafts developed substantial IgM/IgG deposition, complement activation (C4b), and pvWF upregulation, indicating antibody‐mediated rejection (AMR). This suggests either (i) the presence of residual or novel xenoantigens on the graft, such as the hypothesized “fourth antigen” [14, 33, 34, 35, 36], or (ii) failure of the immunosuppressive regimen to prevent a de novo antibody response.

In the later postoperative phase, T cell–mediated rejection (TCMR) became evident despite ATG induction, with CD3^+^ T‐cell infiltration in portal regions. Innate immune activation was also observed, with extensive macrophage (CD68^+^) and myeloid (CD11b^+^) infiltration in necrotic graft regions, highlighting the contribution of both adaptive and innate immune responses to graft loss.

This study demonstrates that porcine livers with multiple gene edits (GTKO/CMAHKO/β4GalNT2KO/hCD55/hTBM) can prevent the development of an immediate lethal coagulopathy (rapid thrombocytopenia, bleeding, and TMA) after LTX in a pig‐to‐NHP model. However, the failure of the xenografts to synthesize key coagulation factors (factor II, protein C, protein S), along with antibody‐mediated, T cell–mediated, and innate immune cell responses, ultimately resulted in progressive graft dysfunction and limited recipient survival. While the expression of hTBM on the graft mitigates early coagulopathy, its long‐term efficacy appears to be restricted by the absence of functional protein C synthesis by the graft.

These results underscore the need for optimizing (i) donor genetic modification, and (ii) the immunosuppressive regimen, and (ii) targeting coagulation factor replacement to retain optimal coagulation function.

Although limited by a small sample size and a short observation period, our study provides critical insights into the mechanisms underlying graft failure and establishes a framework for future approaches to improve outcomes in pig‐to‐NHP LXT.

## Author Contributions

Zhongzhou Si and Haizhi Qi conceived and designed the study. Zhongqiang Zhang, Ting Li, Qiang Li, Bin Xie, Yaxun Huang, Jing Luo, Jiequn Li, and Zhongzhou Si performed the transplant surgeries under supervision of Haizhi Qi. Zhongqiang Zhang, Qiang Li, Bin Xie, Yaxun Huang, and Jing Luo contributed to animal care and perioperative management. Xinger Zhao, Shali Wen, Yinchun Zhou, Yong Deng and Yanfang Lu assisted with the surgeries and recipient management. Jianbin Wang and his colleagues provided anesthetic management. Zhongqiang Zhang and Yanyan Jiang performed assays in vitro, data analysis, and figure preparation. Zhongqiang Zhang drafted the manuscript under supervision of David K. C. Cooper, Zhongzhou Si, and Haizhi Qi, and David K. C. Cooper critically revised the manuscript. Dengke Pan provided the genetically modified pigs used in this study. All authors have read and approved the final version of the manuscript.

All authors would like to thank Hui Guo from Institute of Organ Transplantation, Tongji Hospital, Tongji Medical College, Huazhong University of Science and Technology, China, who assisted with histopathological examinations.

Special thanks are extended to Liang Zhou and his colleagues from the Animal Research Institute of Sichuan Academy of Medical Sciences for their assistance with animal care and perioperative management.

The authors gratefully acknowledge the invaluable support from many colleagues at the Second Xiangya Hospital who collaborated with us on this project over the past 5 years.

This study was supported by the Excellent Youth Foundation of Hunan Provincial Natural Science Foundation, China (2022JJ20087); the Scientific Research Project of Hunan Provincial Health Commission, China (202214023205); the Key R&D Program of Hunan Province, China (2022DK2002 and 2024DK2005); the Joint Fund of the National Natural Science Foundation of China (U24A20656); and the National Key R&D Program of China (2024YFC3406800).

## Conflicts of Interest

David K. C. Cooper consults for eGenesis Bio of Cambridge, MA, USA. Dengke Pan is CEO of Chengdu ClonOrgan Biotechnology Co. Ltd, Chengdu, China. The opinions expressed in this article are those of the authors and do not necessarily reflect those of the companies. The other authors of this manuscript have no conflicts of interest to disclose.

## Supporting information




**Supporting Information file 1**: xen70120‐sup‐0001‐FigureS1.TIF

**Supporting Information file 2**: xen70120‐sup‐0002‐FigureS2.TIF
