# Supplementary figures and images for: Expression of Human Thrombomodulin Prevents Early Thrombocytopenia and Thrombotic Microangiopathy in Pig‐to‐Nonhuman Primate Orthotopic Liver Xenotransplantation
Source: Xenotransplantation. 2026 Mar 23;33(2):e70120. doi: 10.1111/xen.70120 (PMC13051420; doi:10.1111/xen.70120)

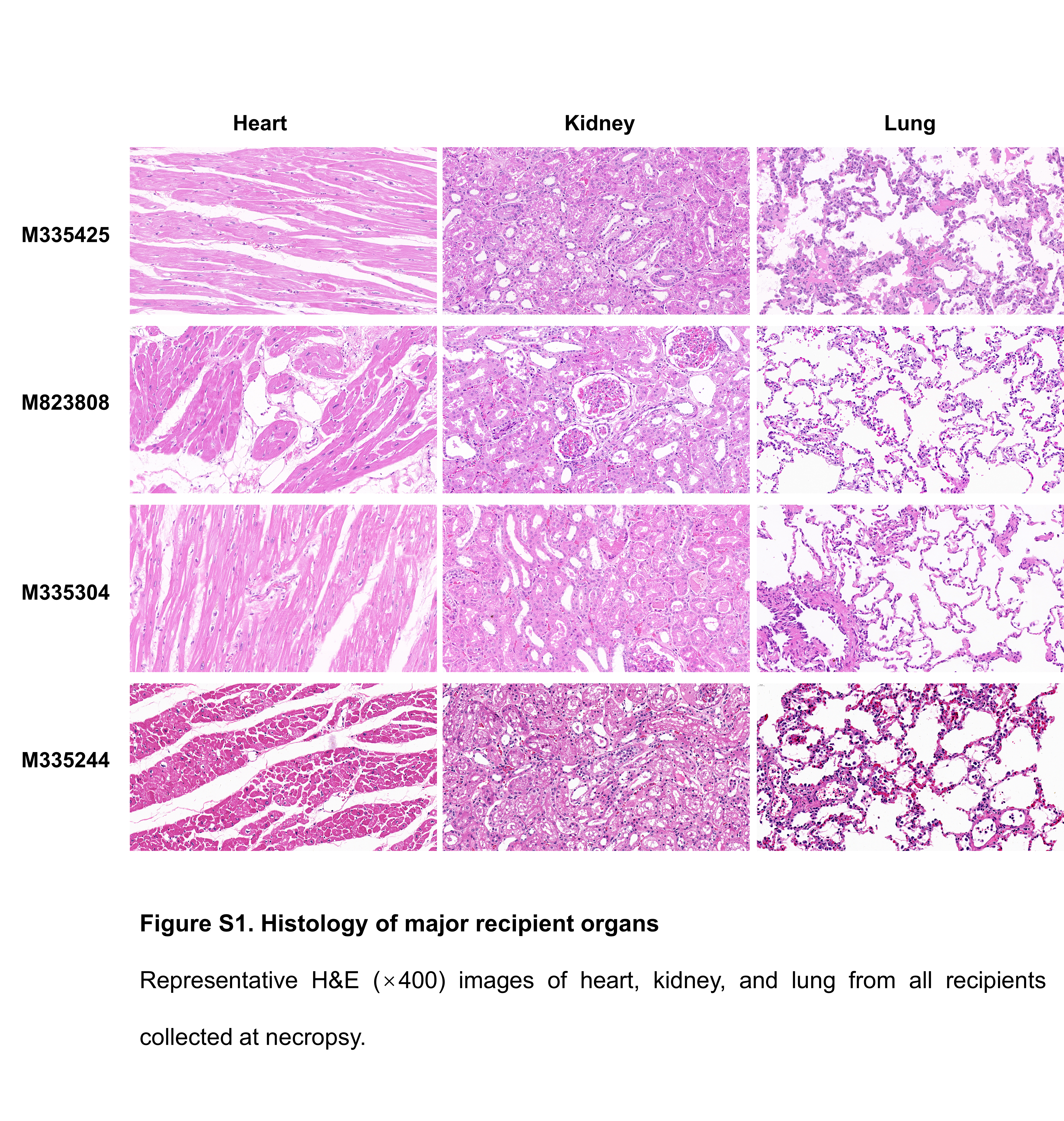

Supplement: Supplementary file 1 — Supporting Information file 1: xen70120‐sup‐0001‐FigureS1.TIF [file XEN-33-e70120-s001.TIF]

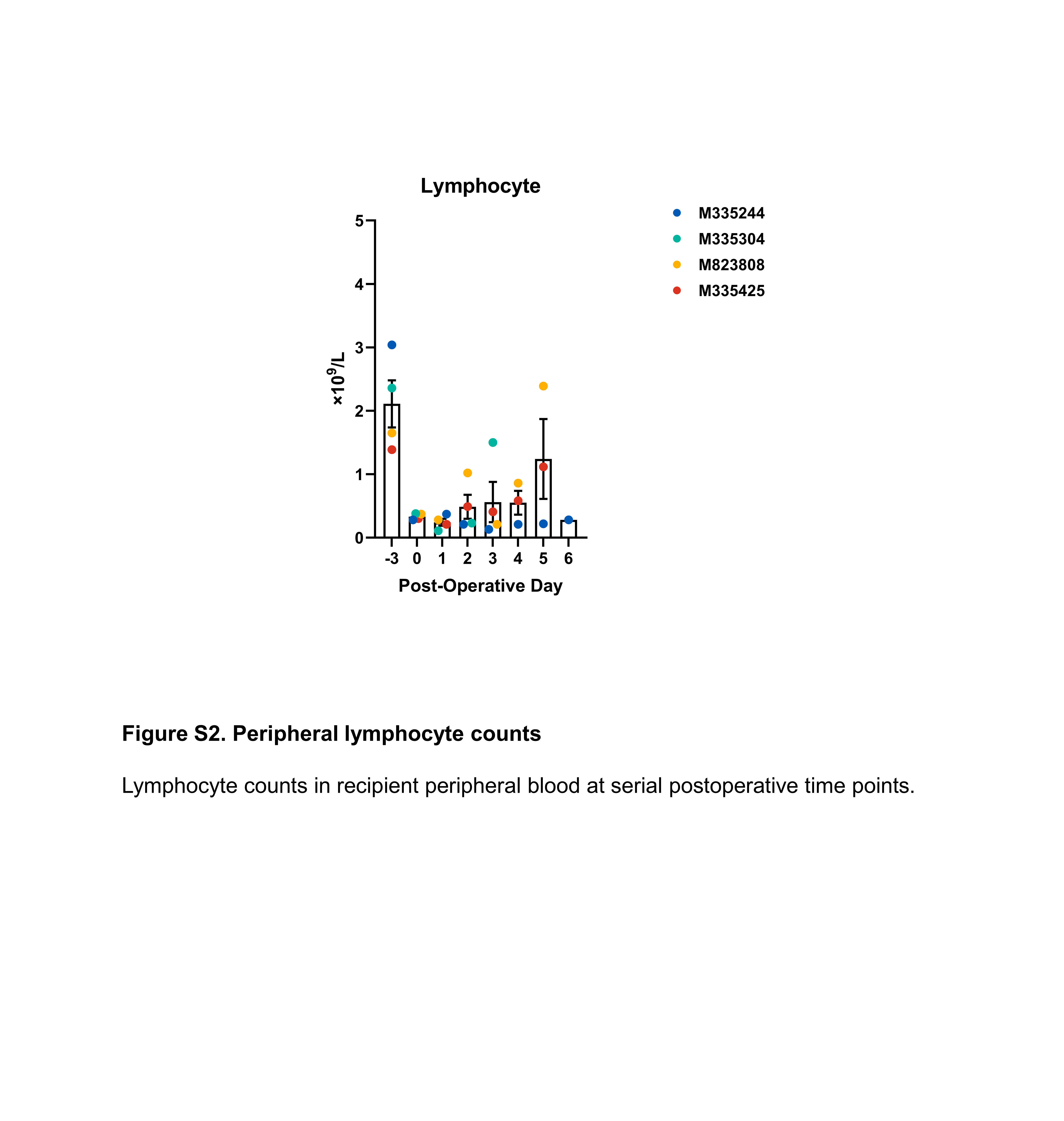

Supplement: Supplementary file 2 — Supporting Information file 2: xen70120‐sup‐0002‐FigureS2.TIF [file XEN-33-e70120-s002.TIF]
